# Factors associated with physician decision making on withholding cardiopulmonary resuscitation in prehospital medicine

**DOI:** 10.1038/s41598-021-84718-4

**Published:** 2021-03-04

**Authors:** Paul Zajic, Philipp Zoidl, Marlene Deininger, Stefan Heschl, Tobias Fellinger, Martin Posch, Philipp Metnitz, Gerhard Prause

**Affiliations:** 1 Division of General Anaesthesiology, Emergency- and Intensive Care Medicine, Medical University of Graz, Auenbruggerplatz 5, 8036 Graz, Austria; 2 Division of Anaesthesiology for Cardiovascular and Thoracic Surgery and Intensive Care Medicine, Medical University of Graz, Graz, Austria; 3 Center for Medical Statistics, Informatics, and Intelligent Systems, Medical University of Vienna, Vienna, Austria

**Keywords:** Health services, Medical ethics, Prognosis, Risk factors

## Abstract

This study seeks to identify factors that are associated with decisions of prehospital physicians to start (continue, if ongoing) or withhold (terminate, if ongoing) CPR in patients with OHCA. We conducted a retrospective study using anonymised data from a prehospital physician response system. Data on patients attended for cardiac arrest between January 1st, 2010 and December 31st, 2018 except babies at birth were included. Logistic regression analysis with start of CPR by physicians as the dependent variable and possible associated factors as independent variables adjusted for anonymised physician identifiers was conducted. 1525 patient data sets were analysed. Obvious signs of death were present in 278 cases; in the remaining 1247, resuscitation was attempted in 920 (74%) and were withheld in 327 (26%). Factors significantly associated with higher likelihood of CPR by physicians (OR 95% CI) were resuscitation efforts by EMS before physician arrival (60.45, 19.89–184.29), first monitored heart rhythm (3.07, 1.21–7.79 for PEA; 29.25, 1.93–442. 51 for VF / pVT compared to asystole); advanced patient age (modelled using cubic splines), physician response time (0.92, 0.87–0.97 per minute) and malignancy (0.22, 0.05–0.92) were significantly associated with lower odds of CPR. We thus conclude that prehospital physicians make decisions to start or withhold resuscitation routinely and base those mostly on situational information and immediately available patient information known to impact outcomes.

## Introduction

Efforts of cardiopulmonary resuscitation (CPR) need to be employed as soon as possible in cases of out-of-hospital cardiac arrest (OHCA) in order to achieve best possible outcomes^1^. For that reason, all links of the so-called chain of survival need to be optimized and complement each other. To provide advanced life support (ALS) interventions as early as possible, most European countries employ systems of skilled ALS providers-both physicians and paramedic personnel-to perform advanced interventions and procedures during prehospital treatment^2^.

With the availability of invasive procedures as early as during on-scene treatment come ethical implications of adequacy of all provided interventions. Ever since the first efforts to provide CPR to patients in cardiac arrest, questions arose about who should be subjected to procedures that sometimes may be perceived as cruel, inadequate and futile^3,4^. This is especially relevant in an ageing society, where the borders of cardiac arrest as an acute, possibly reversible event, and the natural dying process may become progressively blurred^5^.

While interventions of basic life support (BLS) need to be employed without delay to give patients in cardiac arrest chances of survival and meaningful recovery in the first place, the implementation of ALS procedures may seem inappropriate to both witnesses and providers if the chances of meaningful outcome seem to be too low^6^. Current European Resuscitation Council (ERC) guidelines on CPR acknowledge that conundrum and therefore devote an entire section to ethical implications in the provision of CPR^7^.

In Austria, emergency medical services are primarily provided by non-physician staffed ambulances. In cases of life-threatening emergencies—including cardiac arrest—physician-staffed response vehicles or helicopters are also dispatched. Non-physician personnel are legally required to perform CPR in OHCA unless definitive signs of life extinct are found or a legally binding do-not-resuscitate order is in place and immediately available. Prehospital care physicians are trained, entitled and therefore expected to make the decision to perform CPR—usually in the advanced life support algorithm—or withhold this intervention and terminate ongoing efforts. These decisions have to be made quickly, definitively and based upon limited information available in the prehospital setting and within the brevity of time.

### Aim

We aimed to identify the rate at which prehospital care physicians make the decision to start (continue, if already ongoing) or to withhold (terminate, if already ongoing) efforts and interventions of CPR in patients in out-of-hospital cardiac arrest and sought to identify patient-related, process-related and supplemental factors associated with this decision.

## Methods

### Study design, data source and setting

This was a retrospective study using data from the electronic record database of the prehospital physician response system located at the Medical University of Graz, Austria. This ground vehicle-based physician response system covers the east of the greater Graz area in the Austrian state of Styria and provides advanced prehospital medical care for approximately 200,000 inhabitants.

This response unit is staffed by a prehospital care physician trained according to Austrian law and a paramedic assisting the physician. It is available all around the clock on all days of the year and responds to approximately 2000 calls every year upon dispatch by the regional ambulance control centre of Styria. The response unit is not capable of patient transportation and is thus dispatched in conjuncture with local ambulance vehicles provided by the regional ambulance service staffed with emergency medical technicians. These ambulances are usually first to arrive on scene due to closer proximity and higher numbers.

All data documented in clinical routine are collected using an electronic documentation system (MEDEA, iLogs, Austria) and are stored in an electronic database. The dataset is modelled following the MIND3 (Minimaler Notarztdatensatz, Deutsche Interdisziplinäre Vereinigung für Intensiv- und Notfallmedizin [DIVI]) standard set out for documentation in prehospital physician response systems in German-speaking countries. A proprietary export tool for the database is available.

### Patient selection and data extraction

The ethics committee at the Medical University of Graz, Austria (IRB00002556) approved of the study before its conduction and waived the need for informed consent since no additional interventions were performed (decision number 28-387). All used methods and performed analyses were carried out in accordance with relevant guidelines (STROBE statement) and regulations (especially the European General Data Protection Regulation).

Patients were included in this study, if they were attended by the prehospital physician response system for cardiac arrest (defined as NACA score 6 or 7) between January 1st, 2010 and December 31st, 2018. Babies at birth (date of birth equal to date of physician response) were not included. Patients already successfully resuscitated by on-scene personnel upon arrival of the physician-lead team were excluded from the dataset. The export was performed in an anonymised fashion using the proprietary export tool.

**Figure 1:**
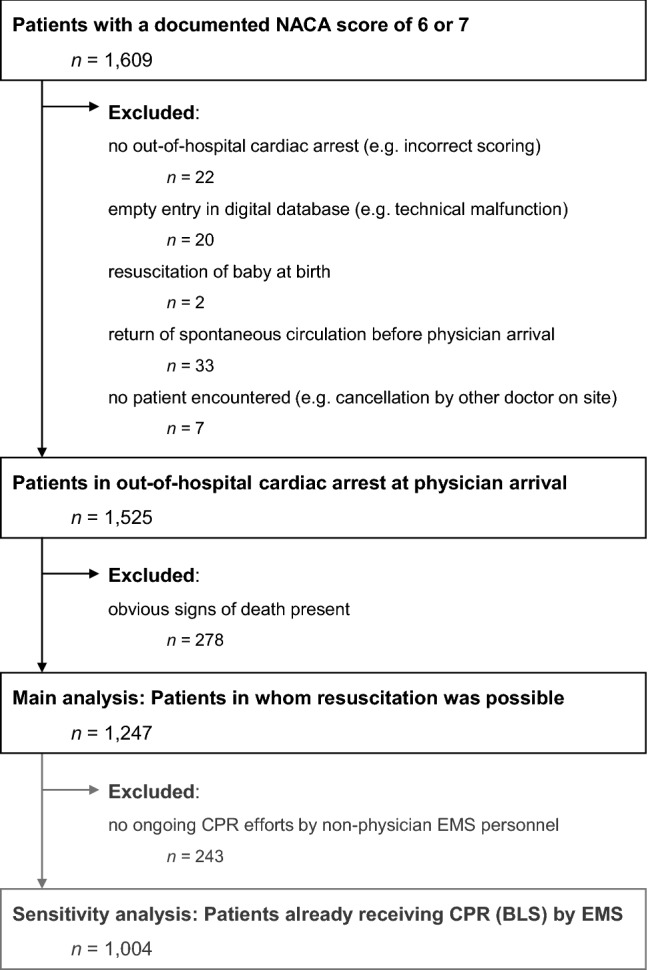
Study flow chart. *BLS* basic life support, *CPR* cardio-pulmonary resuscitation, *EMS* emergency medical services, *NACA* National Advisory Committee for Aeronautics.

The initial query using the described search criteria yielded 1609 results. Applying all exclusion criteria left 1525 patient data sets to be analysed in this study. Of these, obvious signs of death were present in 278 cases, leaving 1247 cases in which the decision to instigate or continue resuscitation efforts had to be made by the 61 prehospital care physicians included in the dataset (Fig. 1).

### Data preparation and variable handling

Data were processed both automatically and manually to comply with the latest Utstein-style for data reporting in CPR research. Patient-related factors (e.g. age, gender, arrest location, witnessed status, bystander response, suspected pathogenesis, …), process-related factors (e.g. physician response time, time of day, year of event, …) and supplemental factors (e.g. comorbidities, standing do-not-resuscitate order, independent status, clinical signs like cyanosis and pupils, …) were derived from the dataset.

Continuous and categorical values already documented in the documentation system were automatically validated for plausibility. Free text fields (such as patient history and clinical examination results) were assessed by two researchers (PZ and PhZ) independently. Cases of disagreement were discussed within the study group (PZ, PhZ, SH and MD); consensus was sought in all cases.

If comorbidities including malignancies were not explicitly documented by the prehospital care physician, they were considered not to be present or not known to the physician. Resuscitation was considered withheld by physicians, if CPR efforts were either not started or terminated within three minutes from arrival at the scene. The full set of variables used and their definitions are presented in the Supplementary materials (Table S1).

### Statistical analysis

Possible influence factors on the decision in question were presented as median and interquartile ranges (IQR) or number and percentages as appropriate. Univariate comparisons were performed using Kruskal–Wallis-test or Fisher’s exact test, as appropriate. For all further analyses only variables with 40 or more cases were included into the model; others were grouped or left out of the models, as appropriate. Single imputation as described by van Buuren and Groothuis-Oudshoorn^8^ was used for missing values. Additionally, univariate logistic regression analyses for all variables under investigation adjusted for anonymous physician ID were performed.

A multivariable logistic regression analysis model with start (or continuation, if already ongoing) of CPR by the physician on scene as the dependent variable and possible influence factors describe above as independent variables was constructed. The model was adjusted for anonymised identifiers of physicians making the decision as random effects.

Patient age was modelled using cubic splines (Figure S1 depicts baseline functions) with confidence intervals calculated according to Hothorn, Bretz and Westfall^9^. To account for multiple testing, simultaneous 95% confidence intervals were reported. Factors for which the simultaneous 95% confidence interval of the odds ratio excludes 1 were considered statistically significant. The impact of the prehospital care physician was described by quartiles of the estimated random effects.

Sensitivity analyses using the same basic model setup were performed to address the handling of missing data: first, categorical variables with “missing” as a separate factor level were used and imputations were used for patient age only; second, fields with missing values were re-coded as the most common expression of the respective variable where feasible. A further sensitivity analysis was conducted in the subset of patients who were undergoing CPR efforts by EMS upon arrival of the prehospital care physician.

C-statistics were used to assess goodness of fit for all models used, where the random effects were set to 0. Ten-fold cross-validation on the physician level was used to correct C-statistics for optimism^10^. All analyses were performed using R version 3.4.2 with packages “lme4”, “mice” and “multcomp”.

### Ethics approval and consent to participate

The ethics committee at the Medical University of Graz, Austria (IRB00002556) approved of the study before its conduction and waived the need for informed consent since no additional interventions were performed (decision number 28-387).

## Results

Patients were primarily male (n = 764, 61%), of advanced age (median 75 years, IQR 63–86, Figure S2) and mostly encountered at home (n = 823, 66%). Only 514 (41%) cases of OHCA were witnessed by bystanders, a further 69 (7%) were witnessed by emergency medical services (EMS) personnel directly. Bystander CPR was provided in 490 (39%) instances. Median physician response time was 11 min (IQR 8–14 min, Figure S3). The majority of patients were in asystole or pulseless electrical activity (PEA) at the first recording of an electric rate rhythm (n = 794, 64%). Resuscitation efforts by non-physician staffed ambulance crews were already ongoing in 1,004 (81%) patients (Table 1).

**Table 1:** Unadjusted system data, patient core data and supplementary patient data according to the Utstein-style in the total patient cohort, in patients without obvious signs of death, in patients in whom resuscitation was either attempted or continued by physicians and in patients in whom it was not; *P*-values for comparisons between the latter two groups using Kruskal–Wallis-test or Fisher’s exact test, as appropriate.

	Total cohort	No obvious signs of death	Resuscitation attempted or continued by physician		
			Yes	No	*P*
*n* of patients	1,525	1,247	920	327	
*System data*					
Time of day (n, %)					0.64
00:00–05:59	213	186	136 (73%)	50 (27%)	
06:00–11:59	522	410	306 (75%)	104 (25%)	
12:00–17:59	464	368	277 (75%)	91 (25%)	
18:00–23:59	326	283	201 (71%)	82 (29%)	
Physician response time [minutes] (median, IQR)	10 (8–14)	11 (8–14)	11 (8–14)	11 (8–15)	0.11
Resuscitation started by EMS before physician arrival (n, %)					< 0.001
No	417	169	19 (11%)	150 (89%)	
Yes	1,020	1,004	848 (85%)	156 (15%)	
Not documented	88	74	53 (72%)	21 (28%)	
*Patient core data*					
Patient age [years] (median, IQR)	74 (62–85)	75 (63–86)	72 (60–82)	85 (73–90)	< 0.001
Gender (n, %)					< 0.001
Male	935	764	614 (80%)	150 (20%)	
Female	565	466	296 (64%)	170 (36%)	
Not documented	25	17	10 (59%)	7 (41%)	
Witnessed arrest (n, %)					< 0.001
Unwitnessed	845	576	373 (65%)	203 (35%)	
Bystander witnessed	519	514	423 (82%)	91 (18%)	
EMS witnessed	69	69	61 (88%)	8 (12%)	
Not documented	92	88	63 (72%)	25 (28%)	
Arrest location (n, %)					< 0.001
Home/residence	1,032	823	589 (72%)	234 (28%)	
Industrial/workplace	16	14	14 (100%)	0 (0%)	
Sports/recreation event	16	14	13 (93%)	1 (7%)	
Street/highway	123	108	91 (84%)	17 (16%)	
Public building	45	42	40 (95%)	2 (5%)	
Assisted living/nursing home	107	100	54 (54%)	46 (46%)	
Educational institution	1	1	1 (100%)	0 (0%)	
Ambulance/medical facility	45	45	42 (93%)	3 (7%)	
Other	25	21	16 (76%)	5 (24%)	
Not documented	115	79	60 (76%)	19 (24%)	
Bystander response (n, %)					< 0.001
None	860	617	381 (62%)	236 (38%)	
Bystander CPR	504	490	425 (87%)	65 (13%)	
Not documented	161	140	114 (81%)	26 (19%)	
First monitored rhythm (n, %)					< 0.001
Asystole	1,056	794	516 (65%)	278 (35%)	
PEA	210	210	179 (85%)	31 (15%)	
VF / pVT	185	185	183 (99%)	2 (1%)	
Not documented	74	58	42 (72%)	16 (28%)	
Pathogenesis (n, %)					0.88
Medical	1,167	1,003	744 (74%)	259 (26%)	
Traumatic	83	61	42 (69%)	19 (31%)	
Drug overdose	8	6	5 (84%)	1 (17%)	
Drowning	6	6	4 (80%)	1 (20%)	
Electrocution	1	0	1 (100%)	0 (0%)	
Asphyxia	74	50	38 (76%)	12 (24%)	
Not documented	186	121	86 (71%)	35 (29%)	
*Supplementary patient data*					
Independent living (n, %)					< 0.001
No	291	254	129 (51%)	125 (49%)	
Yes	1,044	861	690 (80%)	171 (20%)	
Not documented	190	132	101 (77%)	31 (24%)	
Comorbidities (n, %)					
Cardiovascular					< 0.001
No	1,174	926	659 (71%)	267 (29%)	
Yes	351	321	261 (81%)	60 (19%)	
Pulmonary					0.34
No	1,345	1,080	802 (74%)	278 (26%)	
Yes	180	167	118 (71%)	49 (29%)	
Renal					0.77
No	1,449	1,178	870 (74%)	308 (26%)	
YES	76	69	50 (73%)	19 (27%)	
Gastrointestinal / hepatic					0.84
No	1,487	1,215	897 (74%)	318 (26%)	
Yes	38	32	23 (72%)	9 (28%)	
Metabolic					0.72
No	1,415	1,149	846 (74%)	303 (26%)	
Yes	110	98	74 (76%)	24 (24%)	
Malignancy					< 0.001
No	1,403	1,140	862 (76%)	278 (24%)	
Yes	122	107	58 (54%)	49 (46%)	
Neuropsychiatric					0.10
No	1,337	1,078	804 (75%)	274 (25%)	
Yes	188	169	116 (69%)	53 (31%)	
Substance abuse					0.02
No	1,491	1,219	894 (73%)	325 (27%)	
Yes	34	28	26 (93%)	2 (7%)	
Number of comorbidities (n, %)					0.66
0	850	643	470 (73%)	173 (27%)	
1	381	342	259 (76%)	83 (24%)	
2	197	170	127 (75%)	43 (25%)	
3 or more	97	92	64 (70%)	28 (30%)	
Cyanosis present (n, %)					< 0.001
No	904	744	535 (72%)	209 (28%)	
Yes	220	199	167 (84%)	31 (16%)	
Not documented	401	304	218 (72%)	86 (28%)	
Pupils fixed/dilated (n, %)					0.04
No	425	342	269 (79%)	73 (21%)	
Yes	834	711	507 (71%)	204 (29%)	
Not documented	266	194	144 (74%)	50 (26%)	
DNR order (n, %)					< 0.001
No	1,500	1,224	917 (75%)	307 (25%)	
Yes	25	23	3 (13%)	20 (87%)	
suspected suicide (n, %)					0.05
No	1,422	1,185	881 (74%)	304 (26%)	
Yes	103	62	39 (63%)	23 (37%)	

*DNR* do not resuscitate, *EMS* emergency medical services, *IQR* interquartile range, *PEA* pulseless electrical activity, *pVT* pulseless ventricular tachycardia, *VF* ventricular fibrillation.

Resuscitation efforts were either attempted or continued by prehospital care physicians in 920 (74%) cases, whereas they were withheld or stopped in 327 (26%) instances. The unadjusted comparison of these two groups yielded significant differences in system-related factors, core patient factors and supplementary patient factors; results are depicted in Table 1. Results of univariate analyses adjusted for physician ID are presented in the electronic supplement (Table S2).

**Figure 2:**
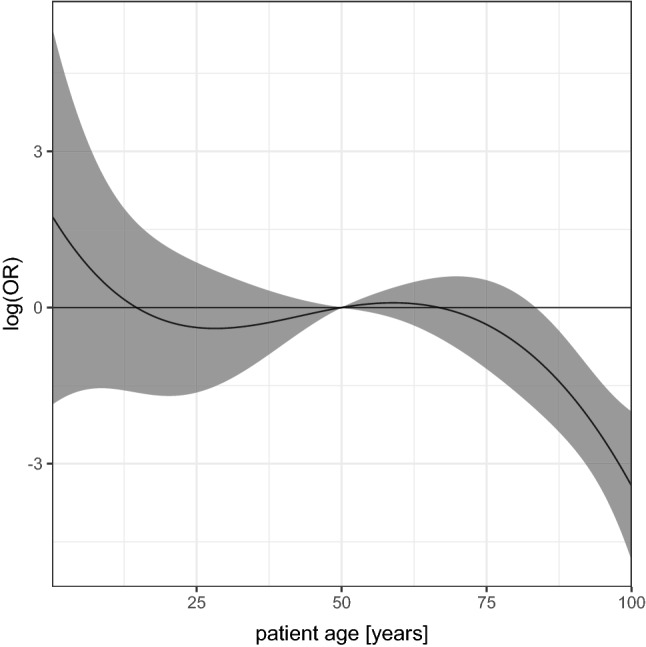
Graphic representation of odds ratios for patient age using cubic splines in main analysis.

In the multivariable logistic regression model, fewer factors were identified as predictors for the decision to start or continue prehospital resuscitation efforts: the physician unit’s response time (OR 0.92, 95% CI 0.87–0.97 per minute), resuscitation started by EMS on physician arrival (OR 60.45, 95% CI 19.89–184.29), patient age (see Fig. 2 for OR), first monitored heart rhythm (OR 3.07, 95% CI 1.21–7.79 for PEA; OR 29.25, 95% CI 1.93–442. 51 for VF / pVT compared to asystole) and known malignancy (OR 0.22, 95% CI 0.05–0.92) (Table 2). Quartiles of estimated random effect for pre-hospital physician were (OR 0.74, 1.36).

**Table 2:** Multivariable logistic regression analysis model for start or continuation, if already ongoing, of CPR by the physician as the dependent variable. Included in the model, but not shown: physician ID as random effects. Cross-validated C = 0.892.

	OR	95% CI	
*System data*			
Time of day (n, %)			
00:00–05:59	1.00		
06:00–11:59	1.38	0.53	3.61
12:00–17:59	1.13	0.42	3.06
18:00–23:59	0.80	0.30	2.13
Physician response time [minutes]	0.93^a^	0.87	0.97
Resuscitation started by EMS before physician arrival	60.45^a^	19.83	184.29
*Patient core data*			
Patient age [years]			
Spline 1	0.03	0.00	35.25
Spline 2	0.14	0.00	98.81
Spline 3	0.10	0.00	32.51
Spline 4	0.01^a^	0.00	0.42
Gender			
Male	1.00		
Female	0.54	0.28	1.04
Witnessed arrest			
Bystander witnessed	1.38	0.67	2.87
EMS witnessed	1.77	0.31	10.32
Arrest location			
Home/residence	1.00		
Street/highway	1.22	0.26	5.75
Public building	2.98	0.21	42.58
Assisted living/nursing home	0.90	0.26	3.06
Ambulance/medical facility	1.15	0.16	8.34
Industrial/workplace, sports/recreation and other	2.08	0.25	17.23
Bystander CPR	1.86	0.87	3.92
First monitored rhythm			
Asystole	1.00		
PEA	3.07^a^	1.21	7.79
VF / pVT	29.25^a^	1.93	442.51
Pathogenesis			
Medical	1.00		
Traumatic	1.94	0.29	13.16
Asphyxia	4.59	0.69	30.64
*Supplementary patient data*			
Independent living	2.35	0.97	5.72
Comorbidities			
Cardiovascular	1.62	0.37	7.12
Pulmonary	0.54	0.14	2.17
Renal	0.87	0.16	4.88
Metabolic	0.36	0.08	1.68
Malignancy	0.22^a^	0.05	0.92
Neuropsychiatric	0.61	0.16	2.40
Cyanosis present	1.60	0.68	3.75
Pupils fixed/dilated	0.77	0.35	1.67
Suspected suicide	0.18	0.02	1.30
Number of comorbidities			
0	1.00		
1	1.15	0.30	4.39
2	1.74	0.17	17.80
3 or more	2.28	0.06	88.88

*CI* simultaneous confidence interval, *EMS* emergency medical services, *OR* odds ratio, *pVT* pulseless ventricular tachycardia, *VF* ventricular fibrillation.

^a^Denotes statistically significant predictors.

Cross-validated C-statistics for this model were 0.892. Results of all conducted sensitivity analyses were similar; cross-validated C-statistics for these models were also comparable. (Table S3–Table S5).

## Discussion

In this study we examine prehospital physicians’ decision-making processes in out-of-hospital cardiac arrest in everyday practice and identify associated factors. We find prehospital physicians readily make the decision to instigate or continue resuscitation efforts without the use of a predefined decision aid. CPR is withheld or immediately discontinued in 26% of all cases of cardiac arrest attended to by physicians in our study. Factors found to be most strongly associated with the decision is whether CPR efforts are already employed by EMS, patients’ age, prehospital physician response time and first monitored heart rhythm.

The proportion of cases in which CPR is either withheld or terminated promptly is similar to the findings of large, multi-national, observational studies on the epidemiology of OHCA and CPR in Europe, which have reported the proportion of CPR attempts to be commenced or continued by EMS at 66% and 62.6%, respectively^11,12^. The underlying decision-making process can be reasonably modelled by multivariable regression analysis and factors associated with this decision can be clearly discerned. This approach allows for comparison of decisions made in everyday practice with results of previous studies that have also tried to understand this decision process, mostly using methods of qualitative and quantitative research based on interviews of and questionnaires sent to medical providers.

One of these studies is a relatively small prospective observational study from 2007 that has found age, previous health status, bystander CPR and adequacy of initial BLS efforts to be the most important influence factors on decision making^13^. Another is a survey in opinion leaders on CPR published in 2016^14^. Here, factors most commonly reported as influential on the decision to commence or withhold CPR prehospitally are patient comorbidity, age, location and knowledge of expressed patient wish against receiving CPR. However, significant differences between countries have been identified, which reduce the applicability of these findings. A further prospective study has been published in 2016, which demonstrated that main factors associated with the initiation of advanced life support are suspected cardiac cause and use of an automated external defibrillator, whereas factors associated with withholding of ALS are higher age, no previous of basic life support, asystole and location in nursing home^15^.

While results of previously published work are thus similar to our study’s findings, they draw a multi-faceted picture of the underlying decision process. In a review of available studies on the subject in 2016^4^, five themes that resuscitation providers seem to incorporate into their decision process have been identified: the arrest event, patient characteristics, the resuscitation scene, resuscitation provider perspective and medicolegal concerns. We thus discuss findings from our study within this framework.

With regards to the arrest event, both time since the event, witnessed status and bystander response have been reported to influence the decision whether to employ resuscitative efforts^16–22^. In this study, only physician response time (as a surrogate for time elapsed since the event and for duration of CPR) has been identified as a predictor for this decision. Neither witnessed status nor bystander response are significantly associated with variation on this decision process in our study. All three aspects (shorter CPR duration, witnessed arrest and bystander CPR) have previously been found to be predictive of improved odds of meaningful survival and recovery^23^.

Multiple studies have demonstrated that initial ECG findings are relevant decision aids for providers^15–22,24^. Our findings confirm this notion, as both PEA and VF / pVT are significant predictors of CPR attempts by prehospital care physicians. A shockable first monitored rhythm has long been known to be associated with higher chances for good outcomes in cardiac arrest, especially in adults^25^. Fixed, dilated pupils are controversial in most studies on decision making^17,19,20^; at least in prognostication after return of spontaneous circulation, fixed, dilated pupils have been found to be relatively reliable^26^. They have not been found to exert a significant effect in our model. The observation of cyanosis has also not been not found to be of significant relevance in this study.

Patient characteristics are hard to assess conclusively within the short timeframe providers have to make decisions on resuscitation efforts. In our study, physicians are clearly less likely to instigate or continue CPR efforts in older patients. This is a more definitive finding than previous interviews and questionnaires would have suggested. In these, respondents have either not uniformly stated that old age would preclude CPR efforts or have indeed disregarded that very notion^16,19,22^. Old age is, however, associated with lower chances of survival and meaningful recovery after out-of-hospital cardiac arrest^27^.

Special attention has been put on patients with pre-existing malignancies^6^. In this study, malignant disease has been shown to predict physicians’ decision making in our regression model. The need for decision making whether CPR should be applied to patients suffering from consuming disease may be considered a failure of advance planning and exploration of patient preference. However, physicians seem to be willing and able to rapidly factor in chances of recovery, length and quality of life to be gained by resuscitation efforts, that all have been shown to be poor^28^, into their decision making. Neither other reported comorbidities nor the number of comorbidities has been found to be associated with changes in the decision process in this study. The presumed pathogenesis of cardiac arrest is also not a significant predictor in our model.

A possible gender effect is suggested by descriptive analyses, but female gender has not been found be associated with lower probability of start or continuation of CPR by physicians in our multivariable logistic regression model. Compared to men, Austrian women are known to have a higher life expectancy, are more likely to be accommodated in nursing facilities with advanced age and more commonly live alone^29^. All of these aspects could lead to differences in baseline patient characteristics. Further research is needed to assess whether this is a gender-driven effect, like recent findings from basic life support research might suggest^30^, or whether there are other factors to be adjusted for, such as in previous studies on outcomes after CPR^31^.

The factor mostly associated with the decision in this study, however, is the resuscitation scene encountered by prehospital physicians. Already ongoing CPR efforts by EMS personnel make resuscitation efforts by physicians drastically more likely. This highlights the situational implications of the decision to be made and is very much in line with findings from questionnaire studies, in which it has been stated that providers perceive the avoidance of (further) delays as vital^4^. Contrarily, time of day or night is not significantly associated with the decision process in this study; there does not seem to be any reluctance to proceed with demanding procedures such as advanced life support due to unfavourable timing in physicians.

Results of the assessment of quartiles of the random effect representing the prehospital physicians actually making the decision to start or withhold resuscitation efforts in our model suggest that resuscitation provider perspective is indeed associated with variation in this decision process. This perspective may vary considerably between providers from different countries, cultures and personal belief sets^14,32^. Adjusted for regional variation, however, resuscitation has been shown to be perceived as inappropriate by providers mostly when a non-shockable rhythm is initially present, when cardiac arrest has not been witnessed, when patients are of older age, and in cases of a “poor” physical first impression^33^. Findings from our study demonstrate that this these factors influencing perception are also associated with decision making.

Medicolegal concerns are perceived as an issue of ever-increasing importance in prehospital care and resuscitation in general. Austrian law allows for definitive decision making by individuals in advance. So-called “patient ordinances” can be signed by adults with capacity following education by a physician and a solicitor; these ordinances constitute do-not-resuscitate-orders or exclude any other medical procedure and are legally binding for both physician and non-physician providers, if they are immediately available. Results from this study show, however, that this option is only rarely used; it may only contribute to decision making processes in rare cases and has thus not been included into the multivariable model.

Some countries, regions and institutions have introduced clinical prediction rules for termination of resuscitation (TOR). Most prominently, the universal TOR rule for BLS providers (BLS TOR) states that resuscitation efforts may be terminated, if no return of spontaneous circulation can be achieved, if the patient has received no shocks and if the cardiac arrest has not witnessed been by EMS personnel^34^. An ALS TOR rule has also been derived from the same patient cohort^35^. It suggests termination of CPR in cases of unwitnessed arrest, no bystander CPR, no shock delivery, and no ROSC. Application if this rule, however, can be associated with drastically higher rates of eventually futile interventions when compared to prehospital decision making by ALS providers^36^.

It is therefore now generally accepted that “resuscitation decisions should be contextualised within overall goals of care” and that “patients receive the right treatments at the right time”^37^. Findings from our study suggest that physicians base their prehospital decision making on factors known to be associated with the achievement of these aims.

### Strengths and limitations

This study is based on a comparably large and detailed dataset, allowing for in-depth analysis into the decision-making process in out-of-hospital cardiac arrest. It is, however, of retrospective design and therefore subject to all limitations that apply to these studies.

Definitive conclusions on causation cannot be drawn from this analysis. This method of analysis, however, is most viable to answer the research question, since prospective randomized trials are not feasible and questionnaires filled in by those making the decisions might be prone to personal and situational biases of respondents.

There is a considerable amount of missing data in the dataset underlying this study. We employ adequate methods of interpolation to allow for the use of these data in our analyses. Even more importantly, these data represent the limited real-world knowledge of prehospital providers who have had to make the decision in question in the first place.

This is a single-centre study in an Austrian physician response system. Its findings can not necessarily be generalized to other regions and other emergency medical systems. The observation period spans over nine years; changes in decision making over this time may have gone unaccounted for.

## Conclusions

Prehospital care physicians readily and routinely make the decision, in which patients suffering from OHCA efforts and interventions of CPR should be start or continued and in whom CPR should be withheld or terminated.

The factor with the strongest association with the decision is situational, i.e. whether CPR efforts are already employed by EMS. Immediately available information on patients, especially their age, are also significantly associated with the decision. Other arrest-related aspects are factored into the decision, especially response time as a proxy for time passed since cardiac arrest and the first monitored heart rhythm. The only comorbidity of immediate relevance appears to be any kind of pre-existing malignancy.

We thus conclude that physicians’ decisions to perform or withhold CPR prehospitally are mostly associated with only a few factors which are immediately available to decision makers and are well known to influence outcomes.

## Supplementary Information

Supplementary Information

## Data Availability

The datasets used and/or analysed during the current study are available from the corresponding author on reasonable request.

## Abbreviations

95% CI: 95 Percent confidence interval
ALS: Advanced life support
BLS: Basic life support
CPR: Cardio-pulmonary resuscitation
DNR: Do not resuscitate
EMS: Emergency medical services
IQR: Inter-quartile range
NACA: National Advisory Committee for Aeronautics
OHCA: Out-of-hospital cardiac arrest
PEA: Pulseless electrical activity
pVT: Pulseless ventricular tachycardia
VF: Ventricular fibrillation
