# Supplementary material for: Factors associated with physician decision making on withholding cardiopulmonary resuscitation in prehospital medicine
Source: Sci Rep. 2021 Mar 4;11:5120. doi: 10.1038/s41598-021-84718-4 (PMC7933171; doi:10.1038/s41598-021-84718-4)
Supplement: Supplementary file 1 — Supplementary Information [file 41598_2021_84718_MOESM1_ESM.pdf]

SUPPLEMENTARY MATERIALS

Withholding Cardiopulmonary Resuscitation in Prehospital Medicine: Factors associated with Prehospital Physician Decision Making

Paul Zajic, MD[1]

Philipp Zoidl, MD[1]

Marlene Deininger, MD[1]

Stefan Heschl, MD PhD[2]

Tobias Fellinger[3]

Martin Posch, PhD[3]

Philipp Metnitz, MD PhD[1]

Gerhard Prause, MD[1]

[1] Division of General Anaesthesiology, Emergency- and Intensive Care Medicine, Medical University of Graz, Graz, Austria

[2] Division of Anaesthesiology for Cardiovascular and Thoracic Surgery and Intensive Care Medicine, Medical University of Graz, Graz, Austria

[3] Center for Medical Statistics, Informatics, and Intelligent Systems, Medical University of Vienna, Vienna, Austria

TABLE OF CONTENTS

List of Variables Used and Their Definitions 2

Spline Base Functions Used 4

Descriptive Statistics: Patient Age 5

Descriptive Statistics: Physician Response Time 6

Univariate Analysis Adjusted for Physician ID 7

Sensitivity Analysis: Categorical Variables With “Missing” as a Separate Factor Level 9

Sensitivity Analysis: Missing Data Imputed With Most Common Values 11

Sensitivity Analysis: Patients undergoing CPR by EMS upon prehospital care physician arrival only .. 13

LIST OF VARIABLES USED AND THEIR DEFINITIONS

Table S 1 List of all Utstein-style variables extracted from the electronic database or derived during data processing. CPR = cardiopulmonary resuscitation, DNAR = do not attempt resuscitation, EMS = emergency medical services, NACA = National Advisory Committee for Aeronautics, PEA = pulseless electrical activity, pVT = pulseless ventricular tachycardia, VF = ventricular fibrillation

Utstein-style variables	variable description	0	1	2	3	4	5	6	7	8	9
id	id as documented in Styrian style										
naca_score	NACA score						survival until hospital admission	dead at the scene/on transport			
patient_dob	patient's date of birth, used for calculation of patient age										
patient_agecategory	patient's age in categories	<2	2-10	11-17	18-45	46-65	66-75	76-85	>85		not documented
patient_age	patient's age										
patient_arrestlocation	site of cardiac arrest	other	home/ residence	industrial/ workplace	sports/ recreation	street/ highway	public building	assisted living/ nursing home	educational institution	ambulance / medical facility	not documented
patient_bystander_response	action by bystanders, relatives, …	none	CPR								
patient_first_monitored_rhythm	first ECG rhythm recorded by EMS	asystole	PEA	VF/pVT	SR						not documented
patient_gender	patient's gender		male	female							not documented
patient_obvioussignsofdeath	signs allowing for recognition of life extinct	none	yes								
patient_pathogenesis	suspected aetiology of cardiac arrest		medical	traumatic	drug overdose	drowning	electrocution	asphyxia			not documented
patient_witnessed_arres	arrest witnessed by bystanders or	no	bystander	EMS							

t	EMS					
process_response_time_physician	duration of physician response, from call to arrival at scene					
process_timeofday	time of day in categories		0h - 6h	6h - 12h	12h - 18h	18h - 24h
supplemental_comorbidities_cardiovascular	pre-existing conditions of cardiovascular nature	no	yes			
supplemental_comorbidities_gastrointestinalhepatic	pre-existing conditions of gastrointestinal nature	no	yes			
supplemental_comorbidities_malignancy	pre-existing malignancies	no	yes			
supplemental_comorbidities_metabolic	pre-existing conditions of metabolic nature	no	yes			
supplemental_comorbidities_neuropsychiatric	pre-existing conditions of neuropsychiatric nature	no	yes			
supplemental_comorbidities_pulmonal	pre-existing conditions of pulmonary nature	no	yes			
supplemental_comorbidities_renal	pre-existing conditions of renal nature	no	yes			
supplemental_comorbidities_substanceabuse	known substance abuse	no	yes			
supplemental_cyanosis	cyanosis present	no	yes			
supplemental_dnacpr	DNAR order or advanced directives available	no	yes			
supplemental_indipendent	patient living independently before arrest	no	yes			
supplemental_pupils_fixeddilated	pupils reported fixed and/or dilated	no	yes			
supplemental_suspected_suicide	arrest suspected to be of suicidal nature	no	yes			
system_resuscitation_attempted_ems	CPR started/continued by EMS	no	yes			
system_resuscitation_attempted_physician	CPR started/continued by prehospital physician	no	yes			

SPLINE BASE FUNCTIONS USED

Figure S 1 Graphical depiction of spline base functions used to model patient age in all analyses

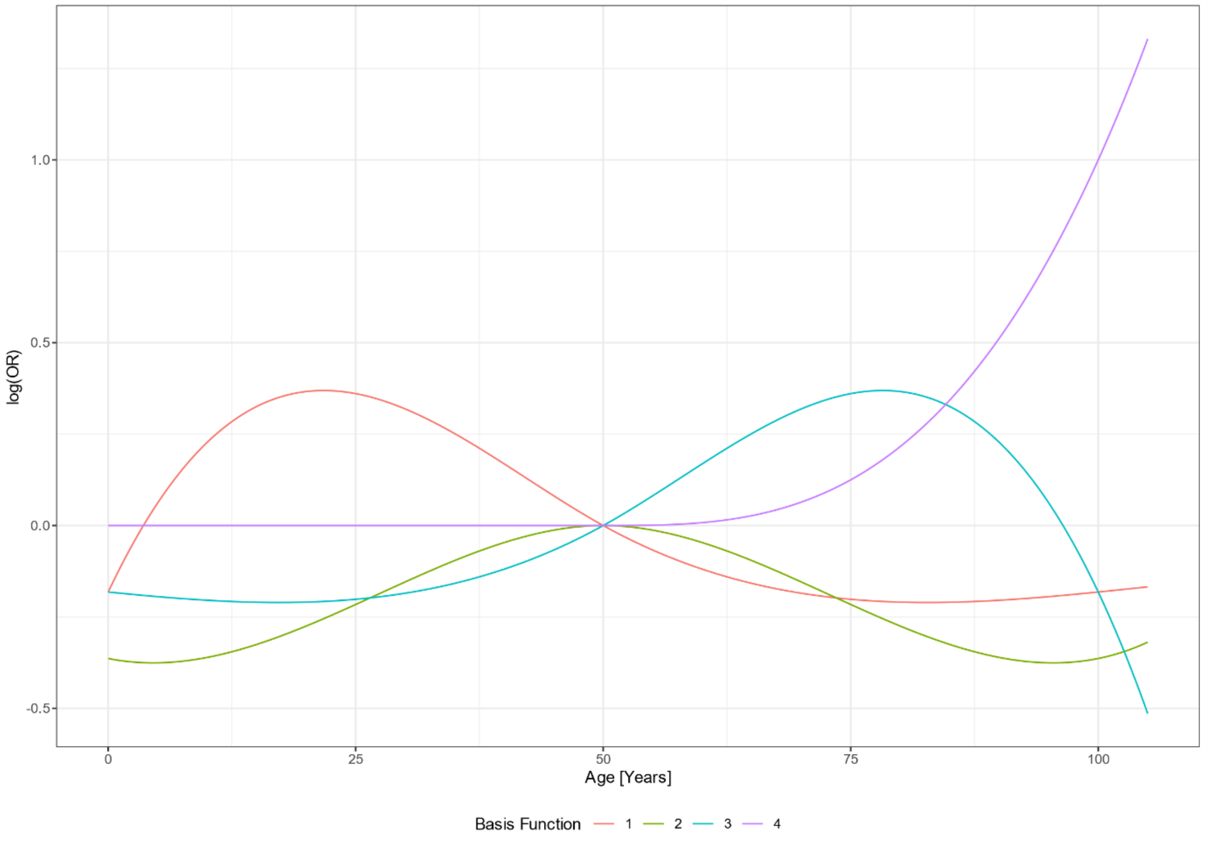

DESCRIPTIVE STATISTICS: PATIENT AGE

Figure S 2 Jitter plot and box plots for patient age in years for patients in whom resuscitation was attempted (or continued) and those in whom it was withheld (or terminated) by physicians.

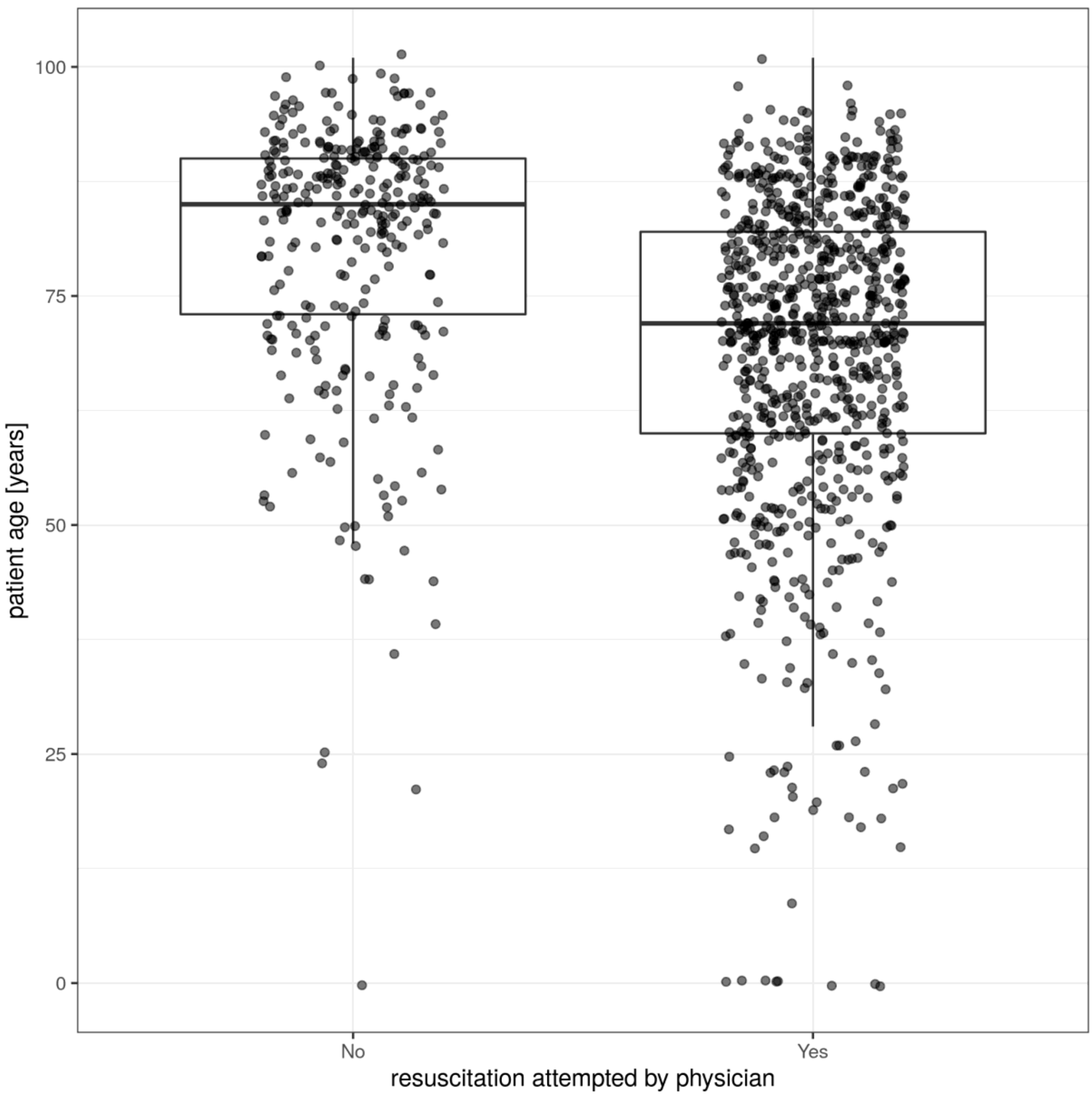

DESCRIPTIVE STATISTICS: PHYSICIAN RESPONSE TIME

Figure S 3 Jitter plot and box plots for physician response time in hours for patients in whom resuscitation was attempted (or continued) and those in whom it was withheld (or terminated) by physicians.

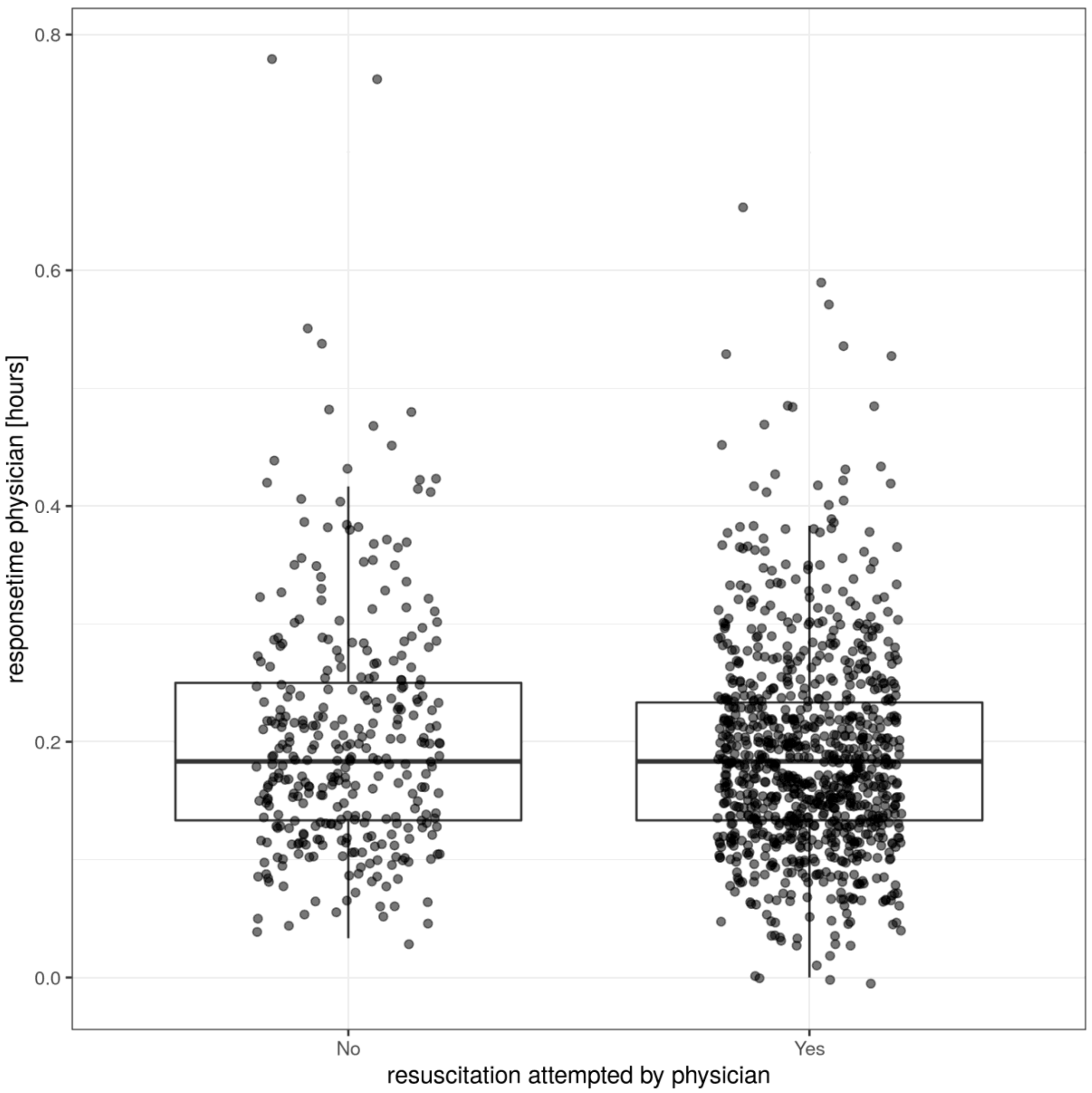

UNIVARIATE ANALYSIS ADJUSTED FOR PHYSICIAN ID

Table S 2 Univariate analyses and C-statistics of variables to be used in the main model; each variable's first coefficient was used as reference; each univariate analysis was adjusted for physician ID. physician IDs not shown in this table.

Variable	OR	95% CI		C
**time of day**				
**00:00-05:59**	1.00			
**06:00-11:59**	1.05	0.69	1.60	0.681
**12:00-17:59**	1.10	0.71	1.68	0.681
**18:00-23:59**	0.96	0.62	1.49	0.681
**physician response time [minutes]**	0.97	0.95	1.00	0.684
**patient gender**				
**male**	1.00			
**female**	0.37	0.28	0.49	0.725
**n/a**	0.50	0.17	1.48	0.725
**patient age category [years]**				
**<2**	1.00			
**2-10**	0.29	0.00	∞	0.746
**11-17**	0.00	1.00	∞	0.746
**18-45**	0.00	0.00	0.00	0.746
**46-65**	0.00	1.00	∞	0.746
**66-75**	0.00	1.00	∞	0.746
**76-85**	0.00	1.00	∞	0.746
**>85**	0.00	1.00	∞	0.746
**n/a**	0.00	1.00	∞	0.746
**first monitored rhythm**				
**Asystole**	1.00			
**PEA**	3.52	2.30	5.54	0.773
**VF / pVT**	52.08	16.27	318.02	0.773
**n/a**	1.27	0.68	2.47	0.773
**witnessed arrest**				
**unwitnessed**	1.00			
**bystander witnessed**	2.54	1.88	3.44	0.719
**EMS witnessed**	4.18	1.99	9.91	0.719
**n/a**	1.28	0.75	2.23	0.719
**pathogenesis**				
**medical**	1.00			
**traumatic**	0.69	0.38	1.27	0.685
**drug overdose**	1.22	0.17	24.59	0.685
**drowning**	2.08	0.30	41.76	0.685
**asphyxia**	0.84	0.42	1.79	0.685
**n/a**	0.73	0.46	1.18	0.685
**arrest location**				
**other**	1.00			
**home/residence**	0.88	0.28	2.35	0.732
**industrial/workplace**	∞	∞	∞	0.732
**sports/recreation**	4.42	0.57	92.80	0.732
**street/highway**	2.03	0.58	6.26	0.732
**public building**	7.96	1.48	61.45	0.732
**assisted living/nursing home**	0.33	0.10	0.97	0.732
**ambulance/medical facility**	4.19	0.88	23.28	0.732
**n/a**	1.10	0.31	3.45	0.732
**bystander response**				
**no bystander CPR**	1.00			
**bystander CPR**	4.38	3.17	6.14	0.748
**n/a**	2.34	1.45	3.89	0.748

**resuscitation started by EMS physician arrival**				
**no**	1.00			
**yes**	71.74	40.52	136.51	0.845
**n/a**	22.81	10.57	52.07	0.845
**comorbidities**				
**cardiovascular**	1.67	1.19	2.36	0.686
**pulmonary**	0.76	0.51	1.13	0.683
**renal**	0.76	0.43	1.38	0.682
**gastrointestinal/hepatic**	0.91	0.41	2.17	0.680
**metabolic**	0.89	0.54	1.52	0.682
**malignancy**	0.33	0.21	0.52	0.696
**neuropsychiatric**	0.65	0.45	0.96	0.689
**substance abuse**	4.04	1.17	25.52	0.684
**number of comorbidities**				
**0**	1.00			
**1**	1.00	0.72	1.40	0.686
**2**	0.94	0.62	1.44	0.686
**3**	0.85	0.47	1.59	0.686
**4 or greater**	0.34	0.13	0.89	0.686
**independent living**				
**no**	1.00			
**yes**	5.77	4.09	8.21	0.751
**n/a**	3.32	1.98	5.68	0.751
**suspected suicide**				
**no**	1.00			
**yes**	0.49	0.28	0.88	0.687
**DNR order**				
**no**	1.00			
**yes**	0.04	0.01	0.14	0.701
**pupils fixed/dilated**				
**no**	1.00			
**yes**	0.59	0.42	0.83	0.688
**n/a**	0.58	0.36	0.94	0.688
**cyanosis present**				
**no**	1.00			
**yes**	1.88	1.21	2.98	0.698
**n/a**	0.65	0.44	0.96	0.698

SENSITIVITY ANALYSIS: CATEGORICAL VARIABLES WITH “MISSING” AS A SEPARATE FACTOR LEVEL

Table S 3 Logistic regression analysis model; dependent variable instigation (or continuation. if already ongoing) of CPR by the physician on scene; model also adjusted for physician ID as random effects. Missing values depicted as separate factor level “n/a”; cross-validated C = 0.882

	OR	95% CI	
system data			
time of day (n. %)			
00:00-05:59	1.000		
06:00-11:59	1.372	0.523	3.602
12:00-17:59	1.189	0.441	3.207
18:00-23:59	0.947	0.355	2.526
physician response time [minutes]	0.926	0.874	0.981
resuscitation started by EMS on physician arrival			
yes	58.812	18.338	188.623
n/a	13.441	2.291	78.863
patient core data			
patient age			
spline 1	3.399	0.038	303.201
spline 2	4.023	0.061	266.024
spline 3	3.430	0.218	54.013
spline 4	0.113	0.013	0.977
gender			
male	1.000		
female	0.529	0.272	1.029
n/a	0.404	0.024	6.756
witnessed arrest			
bystander witnessed	1.521	0.705	3.280
EMS witnessed	1.483	0.240	9.169
n/a	0.805	0.194	3.334
arrest location			
home/residence	1.000		
street/highway	1.269	0.265	6.070
public building	1.509	0.098	23.315
assisted living/nursing home	0.995	0.284	3.492
ambulance/medical facility	1.721	0.186	15.939
industrial/workplace, sports/recreation and other	1.819	0.193	17.111
n/a	1.531	0.332	7.067
bystander response			
bystander CPR	1.629	0.743	3.574
n/a	1.727	0.459	6.502
first monitored rhythm (n. %)			
asystole	1.000		
PEA	3.028	1.163	7.885
VF / pVT	25.710	1.708	386.967
n/a	1.526	0.312	7.458
pathogenesis			
medical	1.000		
traumatic	2.611	0.296	23.066
asphyxia	4.167	0.516	33.662
drug overdose, drowning and n/a	1.371	0.398	4.726
supplementary patient data			
independent living			
yes	2.250	0.865	5.858
n/a	2.064	0.503	8.463

**comorbidities**			
**cardiovascular**	1.397	0.304	6.422
**pulmonary**	0.497	0.116	2.120
**renal**	0.935	0.158	5.531
**metabolic**	0.380	0.076	1.890
**malignancy**	0.245	0.055	1.086
**neuropsychiatric**	0.561	0.135	2.330
**cyanosis present**			
**yes**	1.995	0.696	5.718
**n/a**	1.274	0.375	4.331
**pupils fixed/dilated**			
**yes**	0.664	0.284	1.553
**n/a**	0.704	0.166	2.994
**suspected suicide**	0.205	0.025	1.670
**number of comorbidities**			
**0**	1.000		
**1**	1.273	0.316	5.131
**2**	2.302	0.198	26.782
**3 or more**	2.866	0.060	136.469

Figure S 4 Graphic representation of odds ratios for patient age using cubic splines in the above sensitivity analysis.

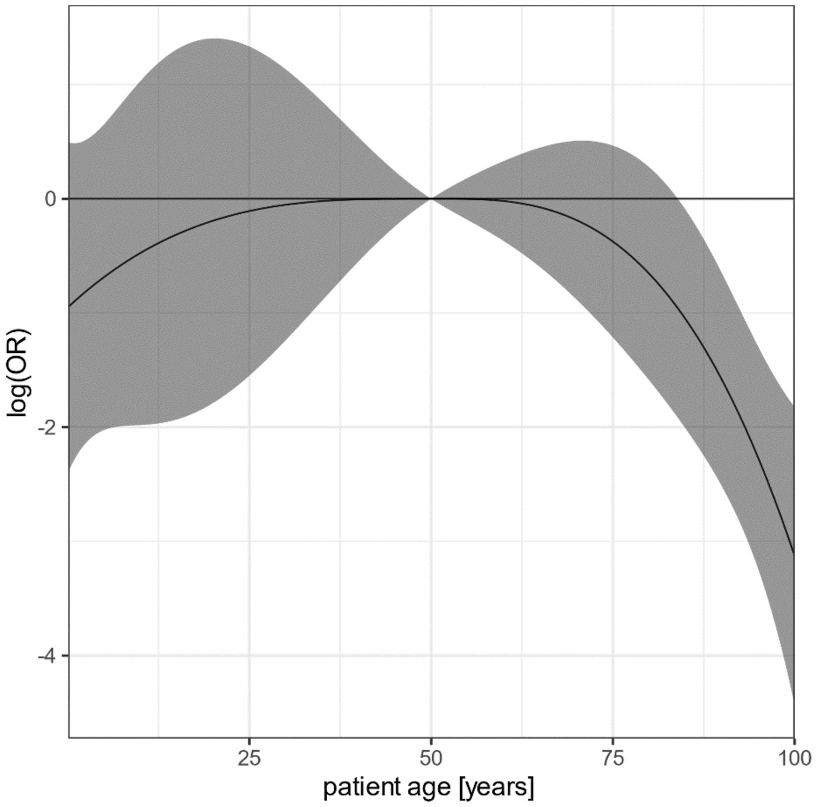

SENSITIVITY ANALYSIS: MISSING DATA IMPUTED WITH MOST COMMON VALUES

Table S 4 Logistic regression analysis model; dependent variable instigation (or continuation. if already ongoing) of CPR by the physician on scene; model also adjusted for physician ID as random effect. Missing values were imputed with their most common expressions. where possible; cross-validated C = 0.884

	**OR**	**95% CI**	
system data			
**time of day (n. %)**			
**00:00-05:59**	1.000		
**06:00-11:59**	1.440	0.569	3.642
**12:00-17:59**	1.269	0.489	3.291
**18:00-23:59**	0.916	0.358	2.341
**physician response time [minutes]**	0.935	0.888	0.985
**resuscitation started by EMS on physician arrival**	50.685	17.078	150.421
patient core data			
**patient age**			
**spline 1**	2.168	0.013	366.294
**spline 2**	0.896	0.010	79.929
**spline 3**	1.444	0.066	31.763
**spline 4**	0.035	0.003	0.422
**gender**			
**male**	1.000		
**female**	0.548	0.291	1.035
**witnessed arrest**			
**bystander witnessed**	1.454	0.720	2.935
**EMS witnessed**	1.566	0.271	9.055
**arrest location**			
**home/residence**	1.000		
**street/highway**	1.153	0.264	5.031
**public building**	1.849	0.124	27.539
**assisted living/nursing home**	1.069	0.317	3.601
**ambulance/medical facility**	1.457	0.168	12.613
**industrial/workplace, sports/recreation and other**	0.958	0.113	8.109
**bystander CPR**	1.530	0.724	3.231
**first monitored rhythm**			
**asystole**	1.000		
**PEA**	3.311	1.329	8.246
**VF / pVT**	17.868	2.011	158.785
**pathogenesis**			
**medical**	1.000		
**traumatic**	1.605	0.196	13.141
**asphyxia**	3.208	0.439	23.461
supplementary patient data			
**independent living**	2.296	0.970	5.432
**comorbidities**			
**cardiovascular**	1.457	0.341	6.227
**pulmonary**	0.501	0.127	1.976
**renal**	0.982	0.185	5.198
**metabolic**	0.403	0.090	1.809
**malignancy**	0.241	0.060	0.974
**neuropsychiatric**	0.624	0.164	2.376
**cyanosis present**	1.197	0.597	2.399
**pupils fixed/dilated**	0.765	0.393	1.490
**suspected suicide**	0.226	0.030	1.707

**number of comorbidities**			
**0**	1.119	0.301	4.155
**1**	1.827	0.184	18.171
**2**	2.504	0.068	92.433
**3 or more**	1.119	0.301	4.155

Figure S 5 Graphic representation of odds ratios for patient age using cubic splines in the above sensitivity analysis.

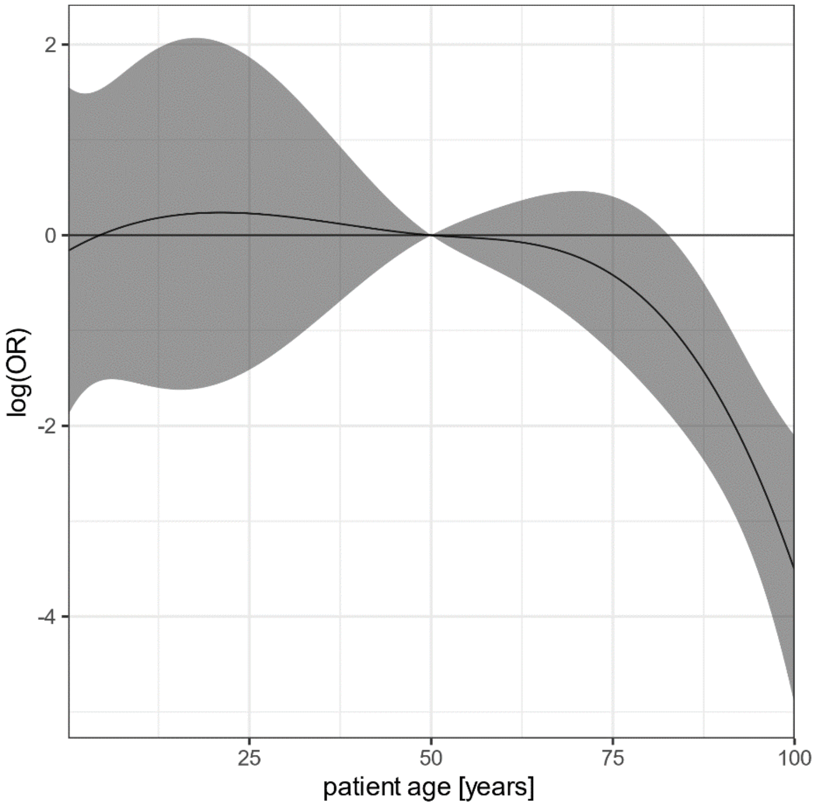

SENSITIVITY ANALYSIS: PATIENTS UNDERGOING CPR BY EMS UPON PREHOSPITAL CARE PHYSICIAN ARRIVAL ONLY

Table S 5 Logistic regression analysis model in a subset of patients who were already receiving cardio-pulmonary resuscitation (mostly basic life support) by emergency medical services upon physician arrival; dependent variable instigation (or continuation. if already ongoing) of CPR by the physician on scene; model also adjusted for physician ID as random effects. Missing values depicted as separate factor level “n/a”; cross-validated C = 0.794

	**OR**	**95% CI**	
system data			
**time of day (n. %)**			
**00:00-05:59**	1.000		
**06:00-11:59**	1.224	0.411	3,652
**12:00-17:59**	1.158	0.375	3,577
**18:00-23:59**	0.857	0.285	2,575
**physician response time [minutes]**	0.005	0.000	0,237
patient core data			
**patient age**			
**spline 1**	1.911	0.006	574,388
**spline 2**	0.709	0.004	115,512
**spline 3**	2.325	0.090	60,333
**spline 4**	0.050	0.004	0,652
**gender**			
**male**	1.000		
**female**	0.523	0.246	1,113
**n/a**	0.151	0.008	2,951
**witnessed arrest**			
**bystander witnessed**	1.392	0.586	3,306
**EMS witnessed**	1.410	0.200	9,923
**n/a**	0.524	0.090	3,050
**arrest location**			
**home/residence**	1.000		
**street/highway**	3.863	0.327	45,593
**public building**	2.064	0.126	33,881
**assisted living/nursing home**	0.987	0.243	4,004
**ambulance/medical facility**	1.619	0.149	17,609
**industrial/workplace, sports/recreation and other**	2.400	0.282	20,461
**n/a**	6.247	0.228	171,210
**bystander response**			
**bystander CPR**	1.816	0.753	4,381
**n/a**	2.289	0.454	11,534
**first monitored rhythm (n. %)**			
**asystole**	1.000		
**PEA**	3.185	1.095	9,270
**VF / pVT**	10.967	0.738	163,020
**n/a**	1.380	0.159	11,994
**pathogenesis**			
**medical**	1.000		
**traumatic**	0.987	0.058	16,866
**asphyxia**	3.814	0.335	43,479
**drug overdose, drowning and n/a**	1.084	0.217	5,429
supplementary patient data			
**independent living**			
**yes**	3.582	1.240	10,343
**n/a**	3.176	0.455	22,182

**comorbidities**			
**cardiovascular**	0.945	0.174	5,136
**pulmonary**	0.418	0.085	2,045
**renal**	0.611	0.083	4,480
**metabolic**	0.305	0.055	1,699
**malignancy**	0.210	0.042	1,060
**neuropsychiatric**	0.576	0.123	2,706
**cyanosis present**			
**yes**	1.782	0.549	5,784
**n/a**	2.292	0.518	10,146
**pupils fixed/dilated**			
**yes**	0.521	0.198	1,371
**n/a**	0.301	0.051	1,766
**suspected suicide**	0.243	0.020	2,908
**number of comorbidities**			
**0**	1.000		
**1**	1.380	0.298	6,380
**2**	2.694	0.181	40,007
**3 or more**	4.421	0.066	297,287
